# Parental Chronic Illness, Internalizing Problems in Young Adulthood and the Mediating Role of Adolescent Attachment to Parents: A Prospective Cohort Study

**DOI:** 10.3389/fpsyt.2021.807563

**Published:** 2021-12-31

**Authors:** Jannike Kaasbøll, Norbert Skokauskas, Stian Lydersen, Anne Mari Sund

**Affiliations:** ^1^Department of Mental Health, Faculty of Medicine and Health Sciences, Regional Centre for Child and Youth Mental Health and Child Welfare (RKBU Central Norway), Norwegian University of Science and Technology, Trondheim, Norway; ^2^Department of Health Research, SINTEF Digital, Trondheim, Norway

**Keywords:** adolescence, attachment to parents, attachment to peers, psychopathological risk, parental chronic illness, internalizing problems, longitudinal, cohort study

## Abstract

**Background:** Parental chronic illness is associated with an elevated risk for developing social-emotional and behavioral problems in children, in particular internalizing symptoms. This study aimed to investigate the associations between parental chronic illness when participants were adolescents and subsequent internalizing symptoms in young adulthood and whether adolescent attachment to parents or peers mediates these associations.

**Methods:** The study used longitudinal survey data from the Youth and Mental Health Study, a cohort study including a representative sample of youth in central Norway assessed in the period from 1999 to 2000 (mean age 14.9 years) and in 2012 (mean age 27.2 years) (*N* = 1,266). The data consist of youth self-reports at both time points. Parental chronic illness was reported by the adolescents, quality of attachment was measured using the Inventory of Parent and Peer Attachment (IPPA), and internalizing problems were assessed in young adulthood by using the Adult Self-Report (ASR). Data were analyzed using parallel mediation analyses, controlling for adolescent sex, parental socioeconomic status, and divorce. In addition, separate analyses were conducted for adolescent girls and boys.

**Results:** The total longitudinal effect was significant for both maternal and paternal chronic illness on internalizing problems in young adulthood. The direct effect on internalizing problems was only significant for maternal chronic illness. Attachment to fathers partially mediated the relationship between maternal chronic illness in adolescence and internalizing symptoms in young adulthood, whereas attachment to both mothers and fathers fully mediated the relationship between paternal chronic illness in adolescence and internalizing symptoms in young adulthood. A separate analysis for girls and boys indicated that the results were only significant for girls. Parental chronic illness did not play a significant indirect effect *via* attachment to peers on internalizing problems.

**Conclusions:** Identifying protective factors in the pathways between parental chronic illness and mental distress in children could guide measures that promote the well-being of the child and family. The study demonstrates the importance of targeting the entire family in chronic illness care.

## Introduction

An increasing number of people suffer from chronic illnesses (1, 2). Four to 12% of people grow up in a household where a parent has a chronic illness (3–6). A few studies have reported positive outcomes concerning parental chronic illness, strengthened interpersonal relationships and increased maturity among children (7, 8). Many more studies have indicated that parental chronic illness increases the risk of reduced family functioning and social-emotional and behavioral problems in children and adolescents (5, 9–11). The risk of adverse outcomes in offspring may increase with the duration and severity of the parent's illness (5, 10). Outcomes for children with chronically ill parents may differ and depend on the sex of the child and the parent (12). Chronic maternal illnesses were associated with poor developmental outcomes, particularly for daughters (10, 12, 13). Paternal chronic illness has been less investigated than maternal chronic illness, and the results by large indicated no significant associations with adolescents' adjustment (10, 12, 13).

Adolescents whose parents are chronically ill are significantly more likely to show internalizing problems (i.e., anxious, depressed, and withdrawn behaviors and somatic complaints) than other children (5, 8, 14–18). While in the general population, adolescent females show more internalizing symptoms than males (19–21), a meta-analysis found that sex differences regarding internalizing problems in children with parental chronic illnesses are negligible (5). Knowledge of the longitudinal effects of parental chronic illness is scarce and inconsistent; however, some studies have indicated that the increased risk of internalizing symptoms persists into adulthood (14, 22). Hence, research into potential mediating and moderating factors in the pathways between parental chronic illness and internalizing symptoms in adolescents as they transition into adulthood is essential for developing effective interventions for families experiencing parental illness (12, 18, 23).

Attachment to parents and peers can both mitigate and increase the risk of future mental health problems, including internalizing problems (24–27). In adolescence, attachment relationships undergo significant changes as adolescents, in general, seek autonomy and independence from their parents, and peer relationships become more important (28). Nevertheless, from early to late adolescence, most teenagers still turn to their parents to solve daily problems (29, 30). In the ecological systems model and family ecological frameworks (18, 23), family-level mediators, including attachment to parents, have been proposed to influence the relationship between parental illness and adjustment in adolescents, as parental illness/disability is one factor that can affect the caregiver's ability to sensitively respond to a child's needs (31). Additionally, potential confounding factors, such as socioeconomic and marital status, should be considered, as they are associated with both parental chronic illness, attachment, and internalizing symptoms in offspring (32–35).

The literature displays mixed results regarding parental chronic illness and attachment in adolescent children. Ireland and Pakenham (36) reported that attachment security to the youth's ill/disabled parent did not predict children's emotional and behavioral outcomes. However, only attachment to the ill/disabled parent was measured, and the authors suggested that the potential negative impact of insecure attachment might be improved by secure attachment to another parent or adult. In contrast, Evans et al. (37) concluded that both insecure attachment and anxiety were more common for children whose mothers had chronic pain, especially for boys, than in the control group (i.e., no maternal chronic pain). Furthermore, Sieh et al. (17) reported that high-quality parental attachment was associated with lower levels of adolescent stress, pointing to a potential protective mechanism in a child's adjustment to parental chronic illness. All these studies measured adolescent self-reported attachment to parents by the “Inventory of Parent and Peer Attachment (IPPA) (38).” A recent study (39) examined the differential predictive powers of the physical and psychological impacts of parental physical illness on adolescent distress. The results indicated that higher quality peer attachment, also measured by the IPPA, was related to lower adolescent distress. In summary, specific implications of parental chronic illness for adolescents' attachment are not fully clear, mainly due to methodological shortcomings: cross-sectional designs and not including attachment to parents and peers in the same model.

The overall aim of the current study was to investigate the association between parental chronic illness in adolescence and subsequent internalizing problems in young adulthood and whether adolescents' attachment to their parents or peers mediates these associations.

## Materials and Methods

### Study Design and Procedures

**Figure 1:**
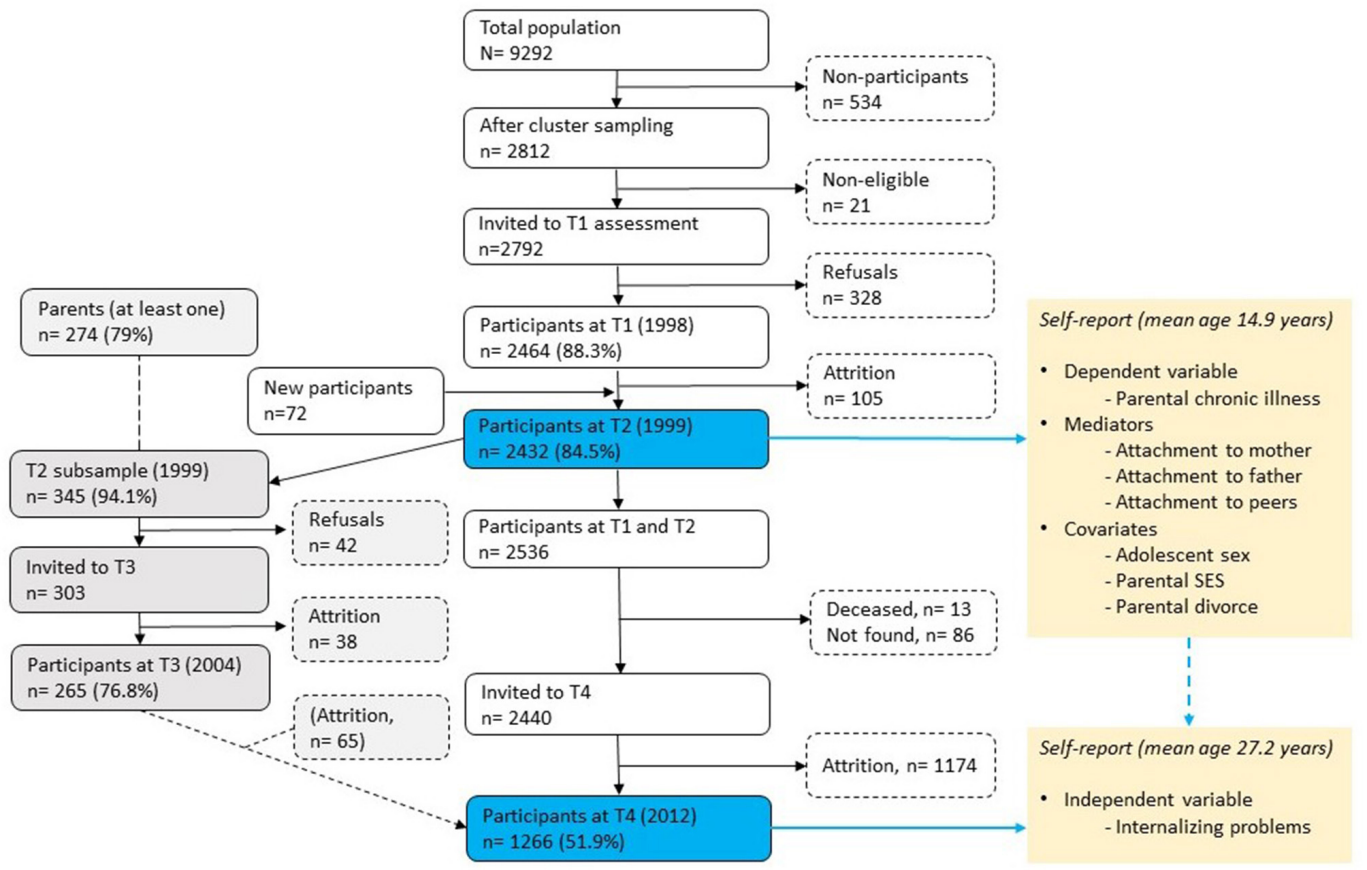
Procedure and flow of participants in the Youth and Mental Health Study. T1 = Time 1, T2 = Time 2, etc. The data used in the current study are highlighted by blue boxes (T2 and T4).

The current study used data from the Youth and Mental Health Study (YAMHS), a population-based, representative (cluster sampling with schools as the units), prospective cohort study that was conducted to investigate risk and resilience factors for mental health conditions, specifically depressive symptoms and disorders, from adolescence to adulthood (40). The first data were collected in 1998 (T1) in two counties in central Norway from 2,464 adolescents (response rate 88.3%, mean age 13.7 years, 50.8% female). The follow-up was conducted in 1999 (T2) (*n* = 2,432, response rate of 87.1%, mean age 14.9 years, 50.4% female). A subgroup of adolescents was assessed at T2 (*n* = 345) with clinical interviews and reassessed in 2005 (T3) (*n* = 265, 70.1%, mean age 20.0 years, 72.5% female) (not part of the present study). The last follow-up (of participants assessed at T1 and T2) was conducted in 2012 (T4) (*n* = 1266, 51.9%, 27.2 years, 56.7% female). The YAMHS is described in more detail elsewhere [see (40)]. Data were collected using questionnaires completed during school time. All waves of data collection were approved by the Regional Committee for Medical Research Ethics in Central Norway [latest approval: REK midt: 2,011/1,454 (T4)]. Since the information on parental chronic illness was not included in the first study wave (T1), the present study used data from T2 and T4 (see Figure 1).

### Measures in Adolescence (T2)

*Parental chronic illness* was measured by using the EASQ (Early Adolescent Stress Questionnaire) (41) that was made for the YAMHS, where the adolescents were asked if they had experienced a multitude of acute or chronic stressors during the last year “[Has this happened to you in the last year (the last 12 months)]?” using yes/no questions. Two of these stressors included parental chronic illness, and the phrasing of the questions in the survey was “Your mother has a chronic disease” and “Your father has a chronic disease.”*Attachment to parents and peers* was measured by the Inventory of Parent and Peer Attachment (IPPA) revised version (38, 42). This instrument was developed to assess adolescents' perceptions of the positive and negative affective and cognitive dimensions of relationships with their parents and peers and how well they serve as sources of psychological security based on attachment theory (43). Each attachment scale comprises three dimensions*: trust* (degree of mutual trust and respect; e.g., “My mother/father/friends respect my feelings”), *communication* (quality and extent of spoken communication; e.g., “I tell my mother/father/friends about my problems and troubles”), and *alienation* (feelings of social isolation, anger, and detachment toward their parents, even though there is a need to approach them; e.g., “My mother/father/friends do not understand what I am going through these days”). The IPPA consists of 25 items each addressing both mothers and fathers, with a Likert scale ranging from 1 to 5 (1 = Never or almost never; 5 = Always or almost always). For space considerations and because earlier research had identified peer attachment as having a lower correlation with maladjustment than attachment to parents (44), nine items were selected from the 25 items constituting the original peer attachment scale that had the highest loading in a factor analysis (45). The total scale was calculated separately for the mother, father, and peers combining these three dimensions, with the Alienation subscale items being reverse-scored. On a dimensional measure, high scores indicate more security in the attachment relationship. The IPPA has shown good psychometric properties in clinical and population-based samples (42, 45–47). The total scales showed good reliability in the sample of this study (T2) (α = 0.90 for attachment to the mother, α = 0.91 for attachment to the father, and α = 0.81 for attachment to peers).

### Outcome Measure in Young Adulthood (T4)

*Internalizing problems* at a mean age of 27.2 years (T4) were assessed with the Adult Self-Report (ASR). The ASR is a part of the Achenbach System of Empirically Based Assessment (ASEBA) taxonomy assessing psychopathology, including adaptive functioning and problems (48, 49). The ASR contains 126 items on problem behaviors that have occurred over the past 6 months. The items are scored on a three-point scale: 0 (not true), 1 (somewhat or sometimes true), and 2 (very true or often true). The ASR consist of eight syndrome scales. The combination of the syndrome scales for Anxious/Depressed (18 items), Withdrawn (9 items), and Somatic Complaints (12 items) comprises the broadband scale for Internalizing problems. For the *internalizing problems subscale*, Cronbach's alpha was 0.93 (T4) in the current study. Higher mean scores indicate higher levels of internalizing problems. The ASR is a well-validated instrument and is used for clinical and research purposes to assess adult psychopathology (48, 50, 51).

*Socioeconomic status (SES)* was measured by the adolescent's report of their mother's and father's occupations, combined with an open question about what their parents did at work, which was classified according to the International Standard Classification of Occupations (ISCO-88) (52) into professional leader, upper middle class, lower middle class, primary industry worker, and manual worker. The father's occupation was utilized unless the adolescent lived with the mother only, in which case the mother's occupation was used. An ordinal scale ranging from 1 (highest) to 5 (lowest) was used. *Parental divorce* was indicated by a yes/no answer to the question “Are your parents divorced?.”

### Statistical Analysis

To examine the indirect effects of parental chronic illness on internalizing problems in young adulthood through attachment to parents and peers, parallel mediation analyses were conducted using SPSS 27 with an added PROCESS macro (available at http://www.afhayes.com) (53). The PROCESS macro uses bootstrapping with the bias-corrected and accelerated (BCa) method to generate the sampling distribution for indirect effects and allows for control variables (54, 55). Their Model 4 (model as a parameter in the PROCESS function) was used for the parallel mediation model, using internalizing problems in young adulthood as the dependent variable, parental chronic illness as the independent variable, and attachment to the mother, father, and peers as the mediators, as illustrated in Figure 1. The analyses were adjusted for adolescent sex, parental SES, and divorce as the control variables. In addition, mediation analyses were conducted separately for adolescent females and males (adjusted for parental SES and divorce) (illustrated in Figures 3, 4). No correction was made for the missing values in the analyses, considering the low proportions missing (2.2-6.5%) among the key study variables. In the analyses, mediation was regarded as significant if the 95% CI for the indirect effect did not include zero.

## Results

### Sample Characteristics

The total study sample (*N* = 1,266) comprised 56.9% (*n* = 720) females. The prevalence of maternal and paternal chronic illness at T2 was 5.2% (*n* = 66) and 4.6% (*n* = 58), respectively. At T2, the participants were, on average, 14.9 (SD = 0.60) years old (range: 13.7–17.0). T4 occurred approximately 12.5 years after T2, and at that time, the participants' mean age was 27.2 (SD = 0.59) years (range: 26.0–28.2).

The distribution of SES was as follows: professional leader (upper class) (10.9%, *n* = 132), upper middle class (32%, *n* = 407), lower middle class (11.8%, *n* = 150), primary industry worker (8.5%, *n* = 108), manual worker (32.6%, *n* = 413), and missing values (4.4%, *n* = 56). A total of 26.5% of the adolescents reported that their parents were divorced. Descriptive statistics for the study variables are presented in Tables 1, 2.

**Table 1:** Descriptive statistics for the study variables (*N* = 1,266).

	***N***	**Min**	**Max**	**Mean**	**SD**	**Range**
Attachment to mother^a^	1,201	1.68	4.68	3.78	0.59	1-5
Attachment to father^a^	1,157	1.48	4.84	3.59	0.62	1-5
Attachment to friends^a^	1,208	1.00	5.00	3.18	0.56	1-5
Internalizing problems^b^	1,266	0.00	56.00	11.90	11.09	0-80

a *Measured by the revised version of the Inventory of Parent and Peer Attachment (IPPA), with high scores indicating more security in the attachment relationship*.

b *Measured by the Adult Self-Report, a part of the Achenbach System of Empirically Based Assessment (ASEBA) taxonomy. Higher mean scores indicate higher levels of internalizing problems*.

**Table 2:** Descriptive statistics for the study variables according to parental chronic illness status (*N* = 1,266).

	**No maternal chronic illness**			**Maternal chronic illness**		
	***N***	**Mean**	**SD**	***N***	**Mean**	**SD**
Attachment to mother^a^	1,108	3.79	0.59	66	3.67	0.57
Attachment to father^a^	1,066	3.61	0.61	62	3.35	0.70
Attachment to friends^a^	1,111	3.17	0.55	64	3.31	0.59
Internalizing problems^b^	1,129	11.63	10.99	66	14.92	13.17
	**No paternal chronic illness**			**Paternal chronic illness**		
Attachment to mother^a^ (T2)	1,106	3.79	0.59	57	3.51	0.60
Attachment to father^a^ (T2)	1,075	3.61	0.62	55	3.31	0.63
Attachment to friends^a^ (T2)	1,109	3.17	0.55	58	3.29	0.66
Internalizing problems^b^ (T4)	1,129	11.67	11.05	58	14.91	13.18

a *Measured by the revised version of the Inventory of Parent and Peer Attachment (IPPA), with high scores indicating more security in the attachment relationship (Range: 1-5)*.

b *Measured by the Adult Self-Report (ASR), a part of the Achenbach System of Empirically Based Assessment (ASEBA) taxonomy. Higher mean scores indicate higher levels of internalizing problems (Range: 0-80)*.

Attrition analysis indicated that the responders at T4 were more frequently female than were the non-responders, and fewer responders had a non-Norwegian ethnicity. The upper middle class was overrepresented among the parents of the responders, while workers were underrepresented (40).

### Mediation Analyses

#### Maternal Chronic Illness

**Figure 2:**
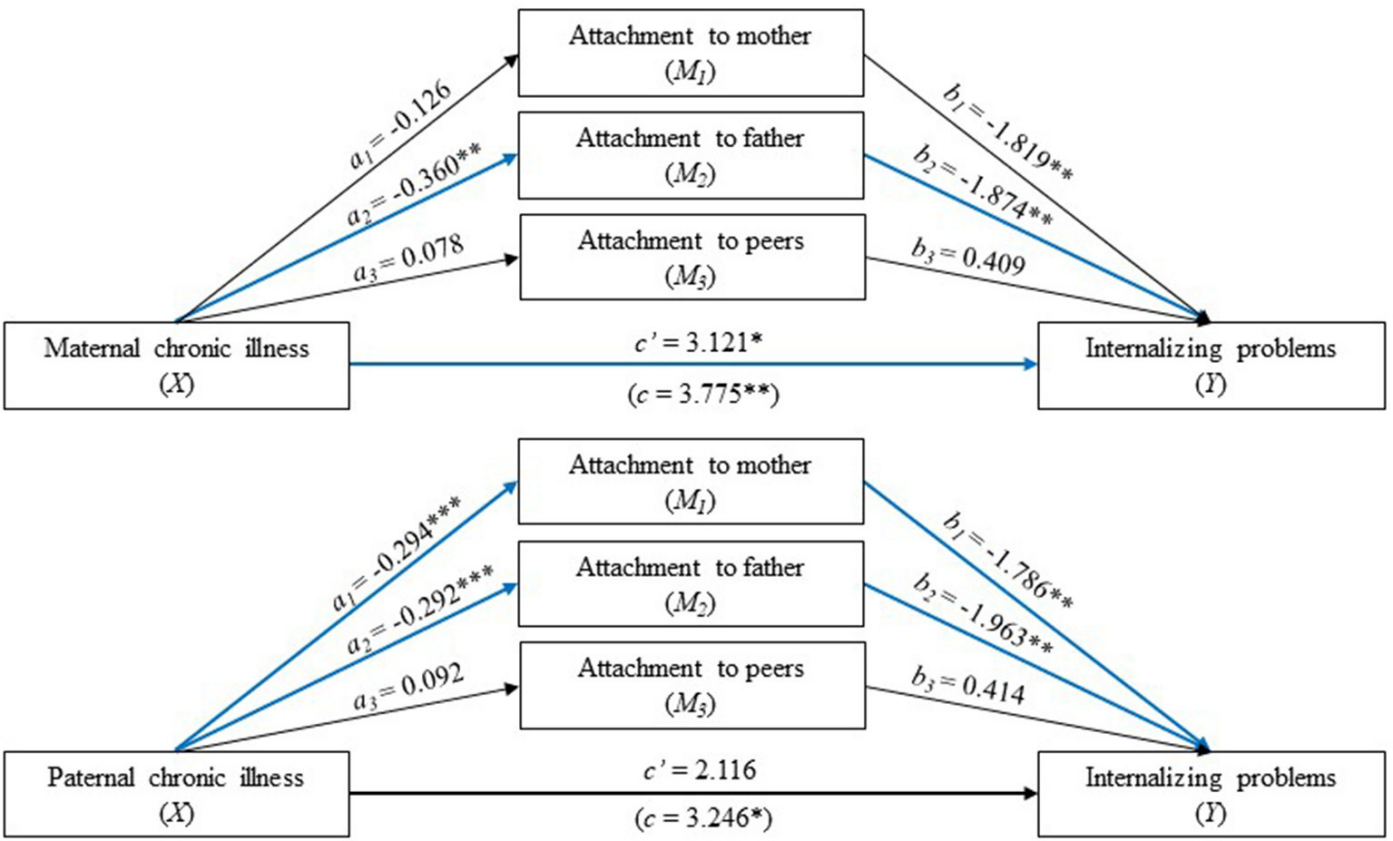
Parallel mediation analysis with parental (maternal above and paternal below) chronic illness status (0 is no and 1 is yes) as the independent variable (X), internalizing problems in young adulthood with a high score indicates high levels of problems (Y), and attachment to the mother, father, and peers as the mediators (a high score indicates secure attachment). The values are unstandardized regression coefficients. a_n_ is the effect of parental chronic illness in adolescence on attachment to the mother, father, and peers; b_n_ is the effect of attachment to the mother, father, and peers on internalizing problems in young adulthood. The analysis was adjusted for adolescent sex and parental SES and divorce. The blue lines show a statistically significant path. *p < 0.05, **p < 0.01, ***p < 0.001.

There was a significant total effect of maternal chronic illness on internalizing problems in young adulthood (coeff. = 3.775, CI: 0.900-6.651) (Table 1). Part of this effect was mediated through attachment to the father (upper part of Figure 2).

First, as seen in Table 3, Figure 2, individuals who reported *maternal* chronic illness in adolescence reported lower levels of secure attachment to fathers than adolescents who reported no maternal chronic illness (coeff. = −0.360, CI: −0.038 to −0.045), and higher levels of attachment to fathers were subsequently related to lower levels of internalizing problems in young adulthood (coeff. = −1.874, CI: −3.180 to −0.567). As seen in Table 3, the 95% confidence interval indicated that the indirect effect through attachment to fathers, holding all other mediators constant, was entirely above zero (0.031-0.906) and hence statistically significant. In contrast, the indirect effects through attachment to both mothers and peers were not significantly different from zero (see Figure 2 for the effects associated with these pathways).

**Table 3:** Path coefficients for the parallel mediation models and separate models for maternal and paternal chronic illness.

	**Maternal chronic illness (*****N*** **=** **1,070)**			**Paternal chronic illness (*****N*** **=** **1,069)**		
	**Coeff**.	**95% CI**	***P*-value**	**Coeff**.	**95% CI**	***P*-value**
Total effect (c)	3.775	0.900 to 6.651	0.010	3.246	0.234 to 6.257	0.035
Direct effect (c')	3.121	0.278 to 5.966	0.032	2.116	−0.875 to 5.106	0.165
a_1_	−0.126	−0.279 to 0.027	0.105	−0.294	−0.453 to −0.135	0.003
a_2_	−0.360	−0.038 to −0.045	0.013	−0.292	−0.461 to −0.123	>0.001
a_3_	−0.092	−0.036 to 0.220	0.159	0.078	−0.056 to 0.211	0.252
b_1_	−1.819	−3.202 to −0.436	0.010	−1.786	−3.174 to −0.398	0.012
b_2_	−1.874	−3.180 to −0.567	0.005	−1.963	−3.272 to −0.654	0.003
b_3_	−0.409	−0.931 to 1.748	0.550	0.414	−0.623 to −0.930	0.257
**Indirect effects** ^**a**^						
Total indirect^a^ effect	0.654	0.073 to 1.310		1.130	0.503 to 1.912	
a_1_b_1_	0.230	−0.053 to 0.647		0.524	0.081 to 1.172	
a_2_b_2_	0.387	0.031 to 0.901		0.573	0.031 to 1.206	
a_3_b_3_	0.038	−0.130 to 0.272		0.032	−0.130 to 0.235	

a *If the 95% CI did not contain zero, the effect was considered statistically significant. The mediation models were adjusted for adolescent sex, parental SES and parental divorce. a_1_-a_3_ are regression coefficients for X_1_-X_3_, respectively. b_1_-b_3_ are regression coefficients for M_1_-M_3_, respectively*.

Adolescents who reported maternal chronic illness also reported higher levels of internalizing problems, even when taking into account the indirect effect of maternal chronic illness through all three dimensions of attachments (coeff. = 3.121, CI: 0.278 to 5.966). Thus, attachment to fathers only partially mediated the relationship between maternal chronic illness in adolescence and internalizing problems in young adulthood. Maternal chronic illness did not display a significant indirect effect *via* attachment to peers on internalizing problems (Table 3).

#### Paternal Chronic Illness

While the total effect of *paternal* chronic illness on internalizing problems in young adulthood was significant (coeff. = 3.246, CI: 0.234 to 6.257), the direct effect was not (coeff. = 2.116, CI: −0.875 to 5.106) (lower part of Figure 2). Overall, the three mediators fully mediated the relationship between paternal chronic pain and internalizing problems (Table 3), indicating that adolescents whose fathers had a chronic illness were more likely to have higher levels of internalizing problems through the experience of lower levels of attachment to their parents and peers.

Two out of the three mediators were found to contribute significantly to the overall indirect effect. Specifically, there was a statistically significant indirect effect of paternal chronic illness on internalizing problems through both attachments to the mother and father (Figure 2). Individuals who reported paternal chronic illness in adolescence reported lower levels of secure attachment to both their mothers and fathers than adolescents who reported no paternal chronic illness. Furthermore, higher levels of attachment to the mother and father were subsequently related to lower levels of internalizing problems in young adulthood. Paternal chronic illness did not display a significant indirect effect *via* attachment to peers on internalizing problems (Table 3).

### Separate Analyses: Adolescent Females and Males

#### Adolescent Females

**Figure 3:**
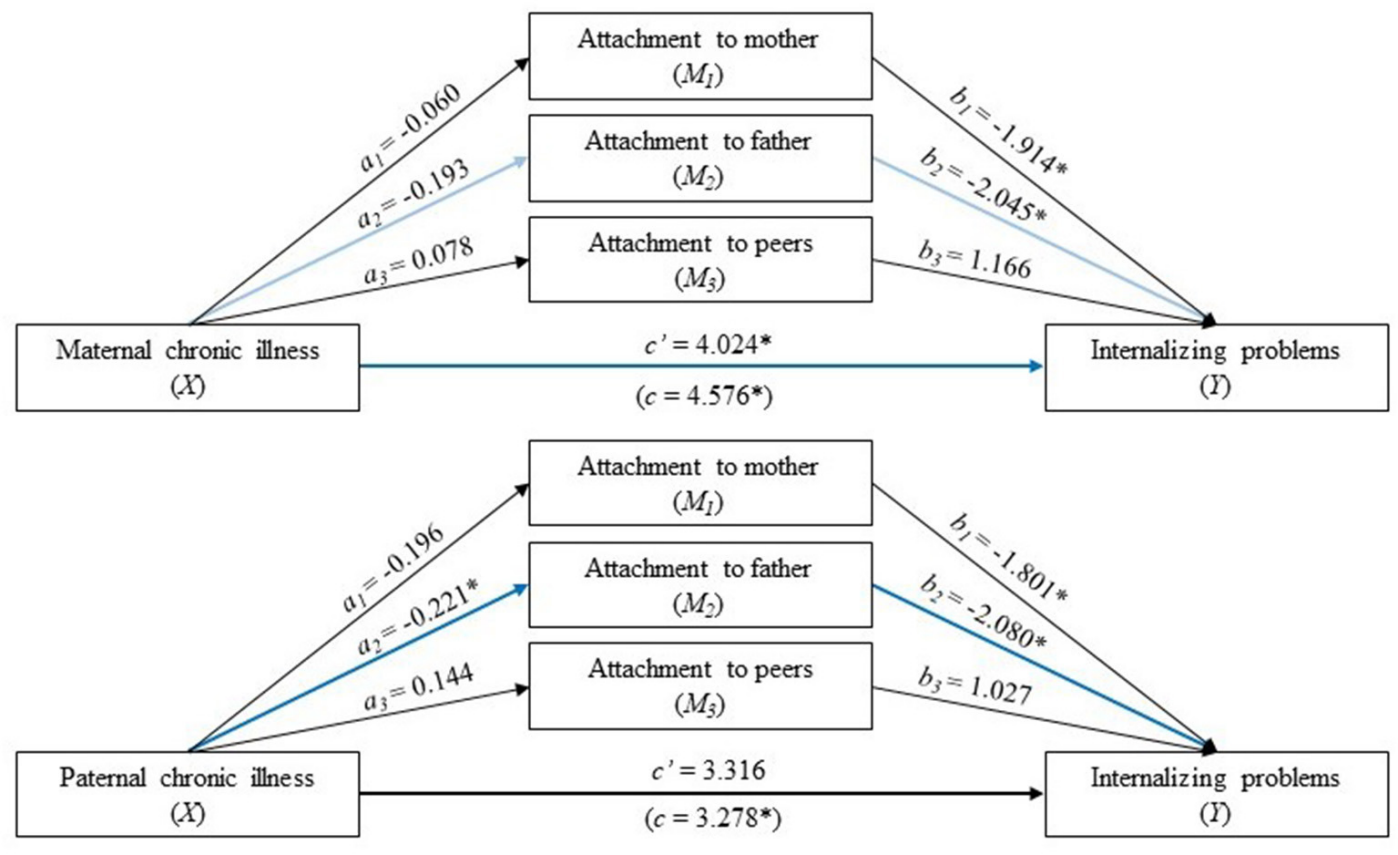
Separate analyses for adolescent females. Parallel mediation analysis with parental (maternal above and paternal below) chronic illness status (0 is no and 1 is yes) as the independent variable (X), internalizing problems in young adulthood with a high score indicates high levels of problems (Y), and attachment to the mother, father, and peers as the mediators (a high score indicates secure attachment). The values are unstandardized regression coefficients. a_n_ is the effect of parental chronic illness in adolescence on attachment to the mother, father, and peers; b_n_ is the effect of attachment to the mother, father, and peers on internalizing problems in young adulthood. The analysis was adjusted for adolescent sex and parental SES and divorce. The blue lines show a statistically significant path (light blue indicates borderline significance). *p < 0.05, **p < 0.01, ***p < 0.001.

For adolescent females, both the total (coeff. = 4.576, CI: 0.770 to 8.383) and direct (coeff. = 4.024, CI: 0.265 to 7.783) effects of *maternal* chronic illness on internalizing problems in young adulthood were significant (Table 4, upper part of Figure 3). In addition, the total indirect effect was statistically significant (coeff. = 0.654, CI: 0.224 to 1.436) (Table 4). There was no significant indirect effect of any of the mediating variables separately. However, attachment to the father was borderline significant (CI: −0.002 to 1.212) (Table 4).

**Table 4:** Separate analyses for female adolescents: Path coefficients for the parallel mediation models, separate models for maternal and paternal chronic illness.

**Adolescent females**	**Maternal chronic illness (*****N*** **=** **611)**			**Paternal chronic illness (*****N*** **=** **612)**		
	**Coeff**.	**95% CI**	***P*-value**	**Coeff**.	**95% CI**	***P*-value**
Total effect (c)	4.576	0.770 to 8.383	0.019	4.278	0.463 to 8.034	0.028
Direct effect (c')	4.024	0.265 to 7.783	0.036	3.316	−0.473 to 7.105	0.086
a_1_	−0.060	−0.260 to 0.141	0.560	−0.196	−0.397 to −0.004	0.052
a_2_	−0.193	−0.400 to 0.014	0.068	−0.221	−0.429 to −0.014	>0.001
a_3_	−0.092	−0.036 to 0.220	0.159	0.144	−0.002 to 0.290	0.054
b_1_	−1.914	−3.677 to −0.151	0.033	−1.805	−3.572 to −0.038	0.045
b_2_	−2.045	−3.749 to −0.342	0.019	−2.080	−3.786 to −0.374	0.017
b_3_	1.166	−0.878 to 3.211	0.263	1.027	0.327 to−1.031	0.327
**Indirect effects** ^**a**^						
Total indirect^a^ effect	0.654	0.224 to 1.436		0.962	0.180 to 1.999	
a_1_b_1_	0.114	−0.245 to 0.589		0.354	−0.011 to 1.007	
a_2_b_2_	0.395	−0.124 to 1.118		0.573	−0.002 to 1.212	
a_3_b_3_	0.044	−0.181 to 0.364		0.032	−0.214 to 0.615	

a *If the 95% CI did not contain zero, the effect was considered statistically significant. The mediation models were adjusted for adolescent sex, parental SES and parental divorce. a_1_-a_3_ are regression coefficients for X_1_-X_3_, respectively. b_1_-b_3_ are regression coefficients for M_1_-M_3_, respectively*.

The total effect of *paternal* chronic illness on internalizing problems in young adulthood was significant (Table 4, lower part of Figure 3), but the direct effect was not statistically significant. There was a statistically significant indirect effect of paternal chronic illness on internalizing problems through attachment to the father. Hence, attachment to the father fully mediated the association between paternal chronic illness and internalizing symptoms in young adulthood.

#### Adolescent Males

**Figure 4:**
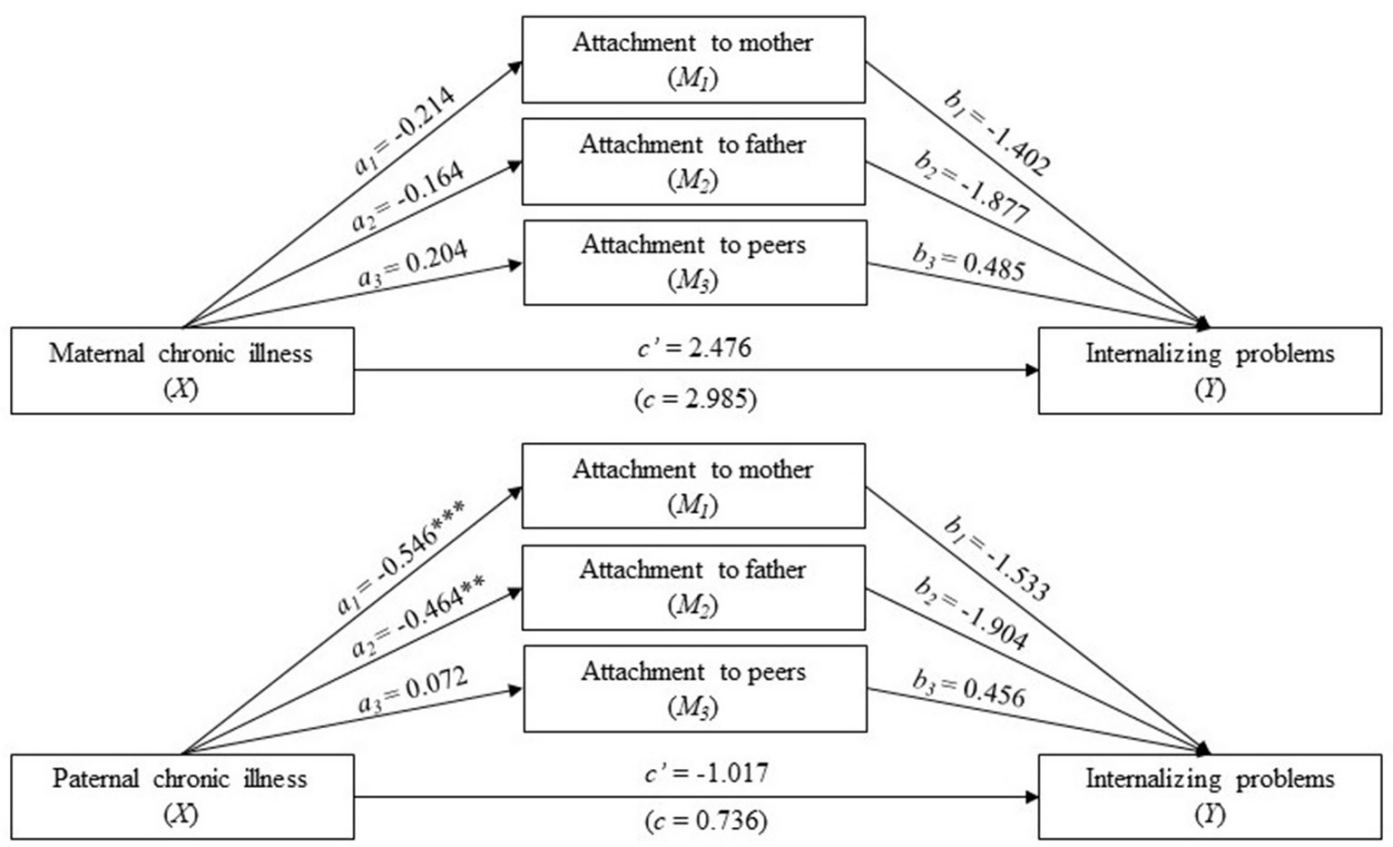
Separate analyses for adolescent males. Parallel mediation analysis with parental (maternal above and paternal below) chronic illness status (0 is no and 1 is yes) as the independent variable (X), internalizing problems in young adulthood with a high score indicates high levels of problems (Y), and attachment to the mother, father, and peers as the mediators (a high score indicates secure attachment). The values are unstandardized regression coefficients. a_n_ is the effect of parental chronic illness in adolescence on attachment to the mother, father, and peers; b_n_ is the effect of attachment to the mother, father, and peers on internalizing problems in young adulthood. The black lines indicate no statically significant paths. The analysis was adjusted for adolescent sex and parental SES and divorce. *p < 0.05, **p < 0.01, ***p < 0.001.

For adolescent males, there were no statistically significant total or direct associations between paternal chronic illness and internalizing problems (Figure 4). Moreover, there was no statistically significant total or direct association between maternal chronic illness and internalizing problems. The total indirect effect was statistically significant (Table 5). However, there were no significant indirect effects of any of the mediating variables separately.

**Table 5:** Separate analyses for male adolescents: Path coefficients for the parallel mediation models, separate models for maternal and paternal chronic illness.

	**Maternal chronic illness (*****N*** **=** **441)**			**Paternal chronic illness (*****N*** **=** **440)**		
	**Coeff**.	**95% CI**	***P*-value**	**Coeff**.	**95% CI**	***P*-value**
Total effect (c)	2.985	−1.465 to 7.434	0.188	0.736	−4.261 to 5.733	0.772
Direct effect (c')	2.476	−1.945 to 6.897	0.272	−1.017	−6.029 to 3.994	0.690
a_1_	−0.214	−0.462 to 0.034	0.091	−0.546	−0.820 to −0.272	>0.001
a_2_	−0.164	−0.436 to 0.108	0.237	−0.464	−0.767 to −0.162	0.003
a_3_	−0.204	−0.041 to 0.448	0.103	0.072	−0.346 to 0.202	0.605
b_1_	−1.402	−3.714 to −0.911	0.234	−1.533	−3.866 to 0.800	0.197
b_2_	−1.877	−3.990 to −0.236	0.082	−1.904	−4.022 to 0.214	0.078
b_3_	0.485	−2.187 to 1.217	0.576	0.456	−2.159 to 1.248	0.600
**Indirect effects** ^**a**^						
Total indirect^a^ effect	0.509	0.575 to 1.636		1.753	0.288 to 3.463	
a_1_b_1_	0.230	−0.359 to 1.224		0.837	−0.661 to 2.544	
a_2_b_2_	0.308	−0.125 to 1.110		0.884	−0.069 to 2.253	
a_3_b_3_	−0.099	−0.751 to 0.410		0.033	−0.573 to 0.536	

a *If the 95% CI did not contain zero, the effect was considered statistically significant. The mediation models were adjusted for adolescent sex, parental SES and parental divorce. a_1_-a_3_ are regression coefficients for X_1_-X_3_, respectively. b_1_-b_3_ are regression coefficients for M_1_-M_3_, respectively*.

## Discussion

Our findings suggest that parental chronic illness serves as both a unique and important risk factor for internalizing symptoms in young adults and that attachment to parents, in particular fathers, acts as a mediator of the effects of parental chronic illness on young adults internalizing problems. These results illustrate the long-term impact of parental chronic illness in adolescence on young adults' functioning 12 years later and highlight the extent to which parental chronic illness can have long-lasting negative effects, particularly for girls.

Our findings add to the very limited body of research suggesting that adolescents who reported parental chronic illness in adolescence had increased levels of internalizing problems in young adulthood. Consistent with previous research (56, 57), the results of the current study indicate that adolescent girls are more vulnerable to internalizing problems if their parents suffer from chronic illness. When girls reach adolescence, they become more vulnerable to interpersonal stress (58, 59), and they may be more likely than boys to take a caregiving role when their parents are ill. Being a young carer has been associated with higher levels of worry and mental health problems for girls than for boys (60, 61), and the caregiving responsibilities for girls persist into young adulthood (62, 63). Kinnunen et al. (8) found that parental illness was associated with lower levels of internalizing problems for boys only. The authors suggested that adolescent boys may cope better than expected with parental illness and that experiencing parental illness may contribute to increased resilience among some boys. However, much less is known about the effect of parental chronic illness on offspring as they emerge into adulthood. Although not statistically significant, it is worth noting that the coefficient for the association between maternal chronic illness and boys' internalizing problems was not substantially changed in the separate analysis. This might also indicate long-lasting adverse effects of maternal chronic illness for boys. The lack of statistical significance for boys could be related to the smaller sample size for boys compared with girls when conducting separate analyses. Hence, future studies should further investigate sex-specific effects regarding the effects of both maternal and paternal chronic illness on girls and boys using larger samples, as well as caregiving roles among emerging and young adults.

Parental chronic illness in the current study was related to lower levels of secure attachment to parents, in particular attachment to fathers. There is no clear consensus on this, possibly due to the methodological shortcomings of previous studies. Our findings concur with Sieh et al. (17) and contradict those of Pakenham and Ireland (36), who found no support for their prediction that lower parent-child attachment security would be associated with poorer youth adjustment. Pakenham and Ireland (36) did not distinguish between attachment to mother and fathers or maternal vs. paternal chronic illness. Moreover, the authors admitted that their study was limited by the small sample size, and several of the scales had low internal consistency, whereas in the current study, the internal consistency of the IPPA scales was satisfactory/very good, and the sample size was sufficient. The findings of the current study add to the existing literature by differentiating the role of attachment to mothers and fathers separately in the context of parental chronic illness and subsequent adjustment of the offspring into young adulthood. To our knowledge, this is the first study to demonstrate that parental chronic illness, through its influences on attachment to parents, increases the risk for internalizing problems during young adulthood.

Attachment to both mothers and fathers fully mediated the relationship between *paternal* chronic illness and internalizing symptoms in young adulthood, whereas attachment to only fathers partly mediated the relationship between *maternal* chronic illness and internalizing symptoms in young adulthood. As suggested by previous studies (10), attachment to one healthy parent may compensate for the potential strain created by chronic illness in the other parent, making the offspring less vulnerable to effects from parental illness. The results of the present study indicate that in the context of *paternal* chronic illness, secure attachments to parents (i.e., both the mother and father) may reduce the risk of internalizing symptoms in young adulthood to a non-significant level. However, when mothers are chronically ill, a more secure attachment to fathers may attenuate the risk, but the risk of increased internalizing symptoms in young adulthood remains significant. Even in industrialized countries with more women in the workforce, mothers are still more likely than fathers to take on childcare responsibilities and maintain family ties (63, 64). Furthermore, sex differences in exchanges of support between parents and grown children are well-documented; mothers exchange more support with offspring (i.e., receiving and providing) throughout adulthood than fathers (63). Similarly, daughters provide and receive more support from parents than sons across adulthood (65). Hence, differences in gender roles and caregiving responsibilities in both parents and children may contribute to different impacts of attachment in the context of maternal and paternal chronic illness. In short, when the mother falls ill, improving attachment to the father only partly compensates for the strain on the adolescent, with long-reaching consequences for mental health. More research is needed to further disentangle such potential mechanisms in the context of maternal compared with paternal chronic illness and the long-term adjustments of offspring.

Parental chronic illness did not display a significant indirect effect *via* attachment to peers on internalizing problems. To some extent, this contradicts the findings of Chen and Panebianco (39), who showed that higher quality peer attachment was related to lower adolescent distress. However, attachment to the parents was not considered in their study. In a parallel mediation analysis, the effect of each mediator was controlled for the other mediators. Hence, this methodological difference could contribute to explaining the contradictory results. Nevertheless, in the current study, although not statistically significant, the adolescents whose parents were chronically ill had slightly higher levels of positive peer attachment than those who did not have chronically ill parents. As mentioned in the introduction, the IPPA peer attachment scale has been noted to have a lower correlation with maladjustment than attachment to parents (44). Thus, other measures/questions on relationships to friends may have provided additional information about this relationship, and future studies should include measures on attachment to parents and peers, as well as other measures on friendship and social support to further explore this association.

### Limitations and Strengths of the Study

The main limitation in the study concerns the sole use of self-reports from the adolescents. Parental chronic illness was defined by adolescents' self-reports, and the phrasing of the question in the survey, asking about “chronic illness of a parent,” does not specify what chronic illness(es) the parents have or about the onset, duration, or severity of the illness. However, Sieh et al. (17) reported that adolescent stress was not related to parental illness type. Moreover, subjective appraisals are likely to be a stronger determinant than more objective characteristics of a stressor in predicting adjustment (66). In a recent Danish study (67), the identification of parental illness from parental hospital diagnoses and self-reports by adolescents was compared. They concluded that both methods have some strengths and limitations. Some adolescents may not have recognized their parent as being chronically ill or were not informed about the illness, which could have resulted in an underestimation of parental chronic illness based on survey data. Nevertheless, the prevalence of parental chronic illness reported by adolescents corresponds with the prevalence estimates of chronic illness reported in other studies (3–6).

Regarding measuring attachment, we relied on a self-report measure that presents the adolescent's perceptions of their relationships with each parent. Only interviews [e.g., the Adult Attachment Interview, AAI (68)] can confirm more accurate internal attachment styles, presumably developed through early years. However, it is conceivable that attachment patterns, secure vs. insecure attachment, respectively, in young people also originate from ongoing social relationships. We, therefore, contend that using the IPPA probably describes not only current relationships but also mirrors enduring attachment structures. Further research would benefit from more in-depth analysis of how the attachment styles has changed over time. In addition, further investigation of attachment, well-being, and quality of life among children whose parents are chronically ill would further add to the existing literature. Furthermore, some of the null findings in the current study, in particular the findings regarding adolescent boys, must be interpreted with care because of the relatively low sample size, when conducting separate mediation analyses for girls and boys (69).

The strengths of the study include the representative population-based sample of adolescents and the longitudinal design, including relatively low attrition rates. Having a stable homogeneous sample and two measurement points allowed for the testing of a truly prospective relationship between parental chronic illness, attachment styles in adolescence, and the development of internalizing problems in young adulthood. In addition, the study also included measures on parental chronic illness and attachment to both fathers and mothers, in contrast to previous studies that mainly focused on mothers (10). Another strength of the study concerns the use of the most widely used and best psychometrically researched attachment (IPPA) and internalizing problems (ASR/ASEBA) self-report instruments.

## Conclusion and Implications

Our findings suggest that parental chronic illness in adolescence serves as both a unique risk factor for the development of young adults' internalizing symptoms and that attachment to parents, in particular fathers, acts as a mediator of the effects of parental chronic illness on young adults' internalizing symptoms. In the context of parental chronic illness, improving attachment between adolescents and parents should therefore be considered when developing policies and practices designed to support parents and their offspring.

The study demonstrates the importance of targeting the entire family in chronic illness care. However, the finding that girls appear to be more vulnerable to parental chronic illness than boys, which is in line with most research on parental illness, demonstrated the importance of investigating sex-specific associations, and it has been suggested that girls whose parents have chronic illnesses may benefit more from stress management than boys (17). Hence, selective interventions specifically aimed at adolescent girls may be valuable in the context of parental chronic illness. Further identification of protective factors in the pathways between parental chronic illness and long-term consequences could guide measures that promote the well-being of the child and family. Additional research should consider the use of structured clinical interviews and multiple sources (e.g., parents, teachers, and observations) as well as family linkage study designs with independent reports from mothers, fathers, and adolescents and/or registry data.

## Data Availability Statement

The datasets presented in this article are not readily available because the public sharing of the data has been restricted by the Regional Committees for Medical and Health Research Ethics following Norwegian law, as YAMHS participants have not given consent to the public sharing of their data. These data are therefore available upon appropriate request to the Norwegian University of Science and Technology, Department of Mental Health, Regional Centre for Child and Youth Mental Health and Child Welfare. Requests to access the datasets should be directed to jannike.kaasboll@ntnu.no.

## Ethics Statement

The studies involving human participants were reviewed and approved by Regional Committees for Medical and Health Research Ethics (REC). Written informed consent to participate in this study was provided by the participants' legal guardian/next of kin.

## Author Contributions

All authors listed have made a substantial, direct, and intellectual contribution to the work and approved it for publication.

## Funding

This article is a part of a large study that was supported by the Research Council of Norway, the Norwegian Council for Mental Health, the Liaison Committee between the Central Norway Regional Health Authority (RHA), and the Norwegian University of Science and Technology (NTNU).

## Conflict of Interest

The authors declare that the research was conducted in the absence of any commercial or financial relationships that could be construed as a potential conflict of interest.

## Publisher's Note

All claims expressed in this article are solely those of the authors and do not necessarily represent those of their affiliated organizations, or those of the publisher, the editors and the reviewers. Any product that may be evaluated in this article, or claim that may be made by its manufacturer, is not guaranteed or endorsed by the publisher.
